# Economic Evaluation for Benign Prostatic Hyperplasia in Iran: Surgical Treatment or Dutasteride

**DOI:** 10.22037/ijpr.2019.111979.13465

**Published:** 2021

**Authors:** Jamaleddin Khedmati, Fatemeh Soleymani, Asiye Moosivand, Saman Zartab, Meysam Seyedifar

**Affiliations:** a *Department of Pharmacoeconomics and Pharmaceutical Administration, Faculty of Pharmacy, Tehran University of Medical Sciences, Tehran, Iran. *; b *Pharmaceutical Management and Economics Research Center, Tehran University of Medical Sciences, Tehran, Iran. *; c *Department of Pharmacoeconomics and Pharmaceutical Management, Faculty of Pharmacy, Shahid Beheshti Medical University, Tehran, Iran.*

**Keywords:** Cost-utility analysis, Dutasteride, Iran, Prostatectomy, Prostatic hyperplasia

## Abstract

Benign prostatic hyperplasia is a common chronic disease that is age-dependent. There are two main types of interventional treatment, transurethral resection of prostate as a gold standard (TURP) and open prostatectomy (OP); also, there are two pharmacological groups for managing BPH: alpha-blockers and 5-alpha-reductase inhibitors (5-ARIs). In this economic evaluation study, one 5-ARIs, dutasteride and two main surgical treatments are compared as alternatives for treating moderate BPH in Iran. A cost-utility study with an Iranian health provider perspective was conducted. Markov model in a cohort of 1000 patients with BPH with annual cycle length and ten years’ time horizon was developed by using MS EXCEL 2013. The effectiveness measure was an improvement in the IPSS score and transformed to the utility. The transition probabilities, utilities and adverse events were extracted from published clinical trials. The direct medical costs were measured in the 2017 US Dollar. One way sensitivity analysis and scenario analysis were conducted.For treating moderate BPH, seventy-year-old men, in the base case scenario, the utility of pharmacotherapy is 18 QALY less than surgery, and the cost of pharmacotherapy is 136,301.1 $ less than surgery. ICER for pharmacotherapy was 7,572.3 $ compared to surgery. In the sensitivity analysis, the model is not sensitive to most variables but the unit cost of dutasteride. Based on scenario analysis conducted for different age groups, pharmacotherapy with dutasteride is preferred to surgery in patients over 60 years of age in Iran. However, for younger adult men between 40-60 years old, surgery is a cost-effective alternative.

## Introduction

Benign prostatic hyperplasia (BPH) is a common chronic disease that is age-dependent. According to epidemiological studies, 75% of men above 70 years suffer from it. This disease is associated with progressive lower urinary tract symptoms (LUTS) (1).

A review of the literature shows that the BPH histological prevalence is similar in the world. However, clinical prevalence varies among countries. According to the Vuichoud study, 23% of patients referred to a urologist were diagnosed with BPH (2). A cross-sectional study in a population of 8446 men over the age of 40 years in Iran shows that the prevalence of BPH in people between 40-49 years old is 1.2% and it increases to 36% in people over the age of 70 years (3).

According to a study performed in the Netherlands, 19% of men between 55-71 years old suffered from BPH and LUTS. They considered BPH as prostate volume > 30 mL and LUTS as IPSS (International Prostate Symptom Score) > 7 (4). The IPSS is based on a validated questionnaire that consists of eight questions: seven questions related to urinary symptoms and one about the quality of life (5). 

The usual symptom associated with BPH is acute urinary retention (AUR). AUR is a situation determined by a sudden inability to pass urine with intense suprapubic discomfort voluntarily. Clinical manifestations of BPH consist of LUTS, incomplete bladder emptying, acute and chronic urinary retention, urinary tract infection, chronic renal insufficiency, hematuria and urosepsis (1).

If the patient does not have polyuria and medical treatment is advisable, the physician may continue treatment based on the following modifiable factors: pharmacotherapy, monitoring of fluid intake especially in the evening and lifestyle changes (6).

There are two pharmacological groups for managing BPH/LUTS: alpha-blockers and 5-alpha-reductase inhibitors (5-ARIs). Dutasteride, which belongs to the 5-ARI group, is a new pharmaceutical product in Iran Market. It acts by inhibiting dihydrotestosterone production; so, it will decrease cell growth and size of the prostate (7). Dutasteride is available in the form of 0.5 mg capsules. It is the second drug in this category approved by the FDA on the market for BPH treatment (7, 8). 

If there is sufficient evidence of blockage and interventional treatment is selected for the patient, TURP (transurethral resection of the prostate) is a gold standard for interventional treatment. A sustained decline in most symptoms and improvement in urodynamic parameters have been reported in longitudinal studies. Studies have shown that patients with bladder-neck obstruction benefit most from obstruction removal (9).

If conservative treatment fails in benign prostatism, open prostatectomy (OP) is the only valuable approach in treating a patient with a large prostate. Satisfying long-term results have been reported on IPSS, post-urinary volume and maximum flow rate by this method. Long-term complications include bladder neck contracture, urethral stricture, and stenosis (6, 10). 

The increasing usage of medicines during the two last decades has transformed the treatment of BPH. There is a drastic decrease in the use of TURP, inpatient hospitalization, and length of hospital stay, and there is a rise in the number of outpatients in the US (11).

As dutasteride has been introduced to Iranian physicians as a new therapeutic alternative, this study is an economic evaluation that compares dutasteride and two main surgical methods (TURP or OP) as alternatives for treating moderate BPH in Iran.

## Experimental

*Methods*

*Model Structure*

A cost-utility study with an Iranian health provider perspective was conducted. The model consists of a decision tree and Markov with annual cycles based on expert opinion. First, in the decision tree patients were divided into two branches of pharmacotherapy or surgery. Then, patients were entered into their specific Markov Model. Markov model was selected because BPH is a chronic disease with multiple clinical events over time. Based on the study of Takayama *et al.*, ten years’ time horizon was selected (12). According to guidelines and experts’ opinions, the population of the study was selected from patients with IPSS = 7-19 (Moderate) who indicated for both pharmacotherapy and surgery (6, 13).

The highest incidence of moderate BPH has been reported in men over 70 years of age (3). Therefore, in our study, the baseline population was the patients over 70 years of age, and the sensitivity analysis was done for other age groups. Seven Percent was applied as the annual discount rate (14), and 3% for utility based on the current rate of global studies (15).

**Figure 1:**
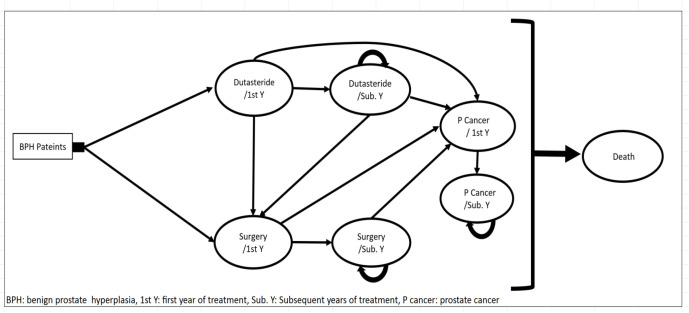
Markov model structure

Markov model consists of seven states presented in Figure 1, including the first year of Dutasteride therapy, other years of Dutasteride therapy, the first year of surgery, additional years of surgery, the first year of prostate cancer, additional years of prostate cancer and death. The first year of Dutasteride therapy is the initial state. Ones who did not have adequate adherence to medication were transferred to the first year of surgery state.

 Others were either transferred to the states of other years of Dutasteride therapy, the first year of cancer or death with certain probabilities. 

In the second year, patients in other years of Dutasteride therapy state remained there or were transferred to the states of the first year of prostate cancer, the first year of surgery, or death. Patients in the state of the first year of prostate cancer entered to the states of other years of prostate cancer or death. Patients in the state of the first year of surgery were transferred to the states of additional years of surgery, the first year of prostate cancer, or death. Patients in the different years of surgery state remained in this state or were transferred to the states of the first year of cancer or death. Patients in the additional years of cancer remained in this state or were transferred to the state of death. In the surgery group, patients were assigned to two types of surgery including TURP and OP, based on their opinions.

It should be mentioned that, because probabilities, utilities and costs of the first year are different from other years, they were considered as two separated states in the model.

*Model Inputs*

*Outcomes*

According to a study by Roehrborn *et al. *in 2004, the effects of Dutasteride on IPSS were followed up to 48 months and the patients’ IPSS indexes were 16.7, 12.3, 11.3, 10.6 at the end of the first to the fourth year, respectively (16). To extrapolate the efficacy of dutasteride for ten years, a linear regression was applied. The first year IPSS was out of range, so it was excluded for prediction. 

Baladi *et al. *study results were used to convert IPSS score to utility (17). Besides, based on the mean age of the patients which were considered to be 70 years, the baseline utility of patients based on the EQ-5D guideline was 0.8 (18). The utilities of patients were obtained by multiplying age-related utility by utilities that were gained from IPSS. The utility reduction from the baseline due to the complications was also considered (19, 20). The mean utility of patients with prostate cancer was supposed to be 0.62 based on the study by Foroughi *et al.* (21). The baseline IPSS was considered 16.7 in both two branches of a decision tree.

The improvement of IPSS after surgery were derived out of Mishriki *et al. *and Hoekstra *et al. *IPSS improvement by TURP was reported from 18.2 ± 7.2 to 8.47 ± 8.2 in 12 years by Mishriki *et al.* and from 13.5 to 6 in 10 years by Hoekstra *et al. *(22, 23). The mean of these two reductions was used for effectiveness, which is equivalent to 8.5 IPSS units. Based on Simforoosh *et al. *the effectiveness of both surgical methods was considered the same (24). Therefor in surgery arm, IPSS decreases from 16.7 to 8.2 after surgery, and the utility of IPSS 8 is determined from Table 1. According to DiSantostefano *et al. *and Armstrong *et al. *studies, the baseline utility was reduced from the likelihood of complications with their incidence rate (19, 25). 

*Costs*

The model accounted for all direct medical costs. For estimation of direct medical cost of the surgery, the below procedure was followed:

Based on experts’ opinion, TURP and OP, 80% and 20% respectively, were performed on patients in Iran, and other methods were restricted to some centers and were rarely used, which could be ignored compared to the above two methods. The number of admission days, para-clinical tests and interventions at the time of admission were determined by consulting the experts and reviewing the patients’ records. The cost of medicines and medical equipment utilized inward and operation room were also taken from hospital records. Tariffs for the hospital stay, diagnostic and therapeutic services were calculated according to the decisions of the Cabinet of Ministers in the public and private sectors. Calculations for various procedures were based on the “Relative Value Unit of Health Care Services in I.R. Iran” book (26).

The price of each Dutasteride capsule based on the manufacturer’s price list is 0.4 USD and it should be taken once daily. Considering the similarity of patient visits, laboratory and diagnostic costs in pharmacotherapy and surgery, and simplifying the model, these costs were not included in calculations. The routine treatment protocol was used to manage the moderate infection and 0.71 USD cost was considered for it. 

We used Foroughi *et al.*, study to calculate the cost of treatment and control of prostate cancer. Accordingly, the mean cost of treatment for each patient with prostate cancer in Iran is 6141 USD. Also, the prices of therapy during the years after the first year were calculated based on the pharmaceutical costs without considering the costs of surgery and radiotherapy which were 2303 USD per patient per year (21). The dollar exchange rate was calculated according to the 2017 rate. According to Zakeri *et al.* study, the proportion of public-private- expenditures based on the ratio of hospital beds in each department was considered 16% to 84% (27).

*Probabilities*

Transition probabilities from one state to another were based on the prevalence rate of the disease in Iran. Probability = (1- e ^-rt^) was used to convert the rate to probability. Where “r” is the instantaneous rate, provided that it is constant for interest time “t”. The probability of death was determined based on the Population Census data from the Statistical Center of Iran and the data published by the Civil Registration Organization for the number of death of different age groups in the year 2011 (28-30). As BPH is not a fatal situation, the likelihood of death in these patients is assumed equal to the probability of death in each age group.

For patients undergoing surgery (OP and TURP), in the first year, the risk of death due to anesthesia and surgery risks (0.002 and 0.001, respectively) was added to the base death probability (22, 24). This amount was not added in the subsequent years after surgery due to the absence of this risk.

The probability of prostate cancer in people with BPH compared to the normal population was considered the same (31). This probability was calculated based on the prostate cancer incidence in Iran and age groups (32). Also, the likelihood of death in prostate cancer patients was determined based on the study of the rate of death in patients with prostate cancer in Iran (33).

The incidence of prostate cancer in Dutasteride users was lower in the first two years after the onset of treatment than in non-drug users based on the REDUCE clinical trial. Also, 25% of the probability of cancer was reduced in these two years (34).

The probability of transition from the state of pharmacotherapy to surgery was considered to be the same as the noncompliance with medication in the first year (19). In other years, it was calculated based on the results of the Cindolo *et al. *study (35). To calculate the cost and utility of surgery, the weighted mean of using TURP and OP methods was used. The probabilities for remaining in each state were obtained by subtracting other possibilities from one.

Side effects of TURP include urethral and meatus stricture, bladder neck stenosis, TURP recurrence, fever, infection, need for transfusion, revision surgery to prevent bleeding, which were considered with a specific probability in a year. The transition probabilities are indicated in Table 2.

## Results

In the base case scenario, the model was run for 1000 patients with moderate BPH aged 70 years and the results show as follows Tables 3 and 4:

According to the results, the utility of using dutasteride is 18 QALY less than surgery, and the cost of this strategy is 136,301.1 USD less than surgery. In other words, the ICER is placed in the third quarter of the cost-effectiveness plane. Accordingly, ICER calculation is essential for the evaluation of alternatives. Hence, ICER calculated for pharmacotherapy was 7,572.3 USD/QALY compared to surgery. Although pharmacotherapy has less QALY than surgery, it can be more cost-saving. The calculated ICER is placed in the acceptable zone based on the willingness to pay (WTP) threshold determined by Iran Food and Drug Association (3,701 USD). This result shows that pharmacotherapy is cost-effective compared to surgery in patients over 70 years of age.

*Sensitivity Analysis*

**Figure 2:**
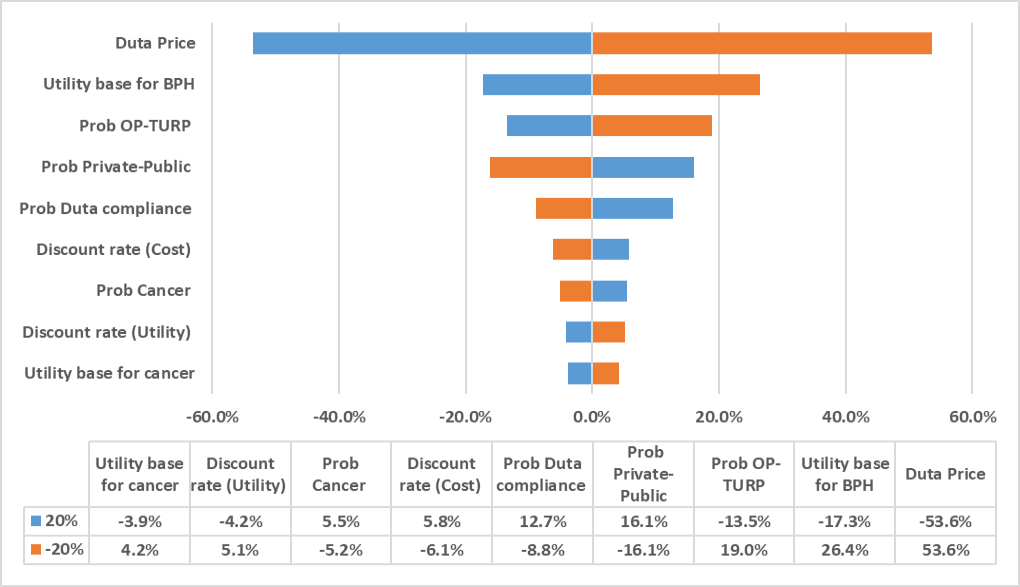
Tornado diagram for one-way sensitivity analysis

To evaluate the effects of various uncertain factors on the results, one-way deterministic sensitivity analysis (DSA) was performed based on changes in probabilities, utilities and costs. In the sensitivity analysis, the following items’ effects were evaluated and the results are presented in the tornado diagram. Figure 2 shows that the model is not sensitive to most variables but the unit cost of dutasteride. 

*Scenario analysis*

*Change in the hypothesis of the effect of dutasteride on cancer prevention*

As noted earlier, based on the results of the REDUCE study, the incidence of cancer in drug recipients decreased to 24% in the first two years compared to the control group. In one scenario, the model was implemented without considering this effect, and the ICER fell from 7,525 to 7,066 USD/QALY, while this decrease did not change the preference for pharmacotherapy.

*Change in TURP/OP ratio*

The assumed ratio based on the opinion of experts was 80-20. However, to assess the effect of change of this ratio, two scenarios were performed with only OP or TURP state, separately. When OP is the only surgical treatment, the results show differences in overall outcomes and pharmacotherapy preference. ICER is not placed in an acceptable zone (2,061 USD), which is probably due to fewer complications of this strategy. In other words, OP is a preferred strategy compared to pharmacotherapy (Table 5).

*Change in the private and public sectors share in the surgery*

Considering all surgical costs according to private sector tariff, ICER is more than $41,424 per QALY indicates a high preference for pharmacotherapy compared to surgery in private sector. This ratio, in the case when purely public-sector costs are calculated, reaches $1,068.4, so it is placed in unacceptable zone. It can be concluded that in this scenario, pharmacotherapy has preference over surgery, in pure private sector (Table 5).

*Changes in the patients’ age*

Since most moderate BPH patients in Iran were at the age of 70 years old (3), this age group was considered the base age group. However, due to the probability of developing the disease from the age of 40, the scenario analysis was also performed for other age groups from forty. In this age group, the likelihood of death was recalculated based on the demographic data of each group, and the baseline utility was considered one for the 40-50-year-old group and 0.94 for the 50-60-year-old group. Therefore, in 40-50 year old patients, the ICER is located in first zone with a value of 4,859.49 cost/QALY, which is above the WTP threshold. Hence, in this age group, surgery was preferred over pharmacotherapy. In the 50-60-year-old group, similar to the previous age group, the surgery was preferred. In the 60-70-year-old group, the results were similar to the baseline model group (70-80 years old), which indicated the preference of pharmacotherapy over surgery in this age group (Table 5).

**Table 1:** Utilities based on IPSS score

**IPSS Score**	**Utility**
1	1
2	0.995
3	0.99
4	0.985
5	0.98
6	0.975
7	0.97
8	0.944
9	0.936
10	0.928
11	0.92
12	0.912
13	0.904
14	0.896
15	0.888
16	0.88
17	0.872
18	0.864
19	0.856
20	0.848
21	0.84
22	0.832
23	0.824
24	0.816
25	0.808
26	0.8
27	0.792
28	0.784
29	0.776
30	0.768
31	0.76
32	0.752
33	0.744
34	0.736
35	0.728

**Table 2:** Transition probabilities

**States**	**Probabilities**	
	**First year**	**Second year**
Medication to Surgery	0.3	0.005
medication to PC	0.000836	0.000836
Medication to Death	Age related	Age related
PC to death	Age related +0.18	Age related +0.18
Surgery to PC	0.0011	0.0011
Surgery to Death	Age related +0.0012	Age related +0.0012

**Table 3:** Costs of two alternatives

**State**	**Cost**	**Description**	**Complication**
First year of Dutasteride therapy	146.36	Medication	
Other years of Dutasteride therapy	146.36	Medication	
First year of Surgery	840.96	Surgery Management of Complications	Bladder neck contractures Secondary endoscopic intervention Cystitis Epididymitis Postoperative fever Bleeding requiring transfusion Urethral strictures Meatal stenosis Surgical revision due to severe bleeding
Other years of Surgery	9	Management of Complications	Bladder neck contractures Secondary endoscopic intervention
First year of Prostate cancer	6,141.00	Surgery Radiotherapy Medication	
Other years of Prostate cancer	2,303.00	Medication	

**Table 4:** Economic evaluation result

**Treatment**	**Total Cost ($)**	**Total QALY**	**Inc. Cost**	**Inc. QALY**	**ICER**
Pharmacotherapy	667,812.6	3193	136,301.1	18	7,572.3
Surgery	804,113.66	3211			

**Table 5:** The results of scenario analysis

	**Incr. Cost**	**Incr. QALY**	**ICER**
Base case	(136,301.06)	(18.11)	7,525.46
surgery just public cost	(19,355.60)	(18.11)	1,068.66
surgery just private cost	(750,264.75)	(18.11)	1,423.65
without chemoprevention effect	(132,357.31)	(18.73)	7,066.31
just open surgery	(162,496.31)	(78.81)	2,061.85
just TURP	(129,752.25)	(2.94)	44,175.24
40-50 years old (QALY base 1)	101,229.75	20.83	4,859.49
50-60 years old(QALY base .94)	64,214.56	12.33	5,209.32
60-70 years old (QALY base .88)	(10,417.85)	(1.40)	7,453.56

## Discussion

To the best of our knowledge, this is the first study of cost-effectiveness for the treatment of BPH with ten years of medical treatment or immediate surgery in Iran, using QALYs to measure outcomes and resource use in association with a randomized clinical trial. The results suggest that surgery in a younger group of patients is more cost-effective than management with pharmacotherapy. Dutasteride is also preferred in patients 60 and older years old. This investigation was done to get a more comprehensive concept about the rational decision.

Despite a thorough search in databases, we could not find any study, which compared dutasteride with surgery. However, several economic evaluation studies compared medical therapy with different types of surgery. In one cost-effectiveness analysis, minimally invasive treatment, alpha-blockers therapy and TURP were compared in 65 years old patients with moderate to severe BPH.

The result showed that minimally invasive therapy was more cost-effective than medical treatment and dominant over TURP. Also, medical treatment is the preferred strategy in comparison with TURP (36). In the other economic evaluation with over 20-year time horizon watchful waiting, microwave thermotherapy (TUMT), TURP, and combination of α-blockers and 5-ARIs, were compared in patients with moderate and severe BPH separately. The results demonstrated that α-Blockers and TUMT were cost-effective for treating moderate symptoms, and TURP was the most cost-effective treatment for severe symptoms from a payer perspective (19).

It is important to understand the trends in utilization that affect the treatment patterns for BPH and the consequent cost-effectiveness. While medical therapy is a common first-line treatment option for mild-to-moderate voiding symptoms, TURP has been the main form of BPH surgical treatment for many years as a standard for improvements in urinary function to which other therapies compared. Pharmacotherapy and technical interventions for BPH have continued to evolve as clinicians learn more about the disease (37).

As it has been mentioned, although the model was most sensitive to the acquisition cost of dutasteride, minimal effects were observed for other key parameters. In scenario analysis of different age groups, the results were affected by changing the age of patients. The DSA indicated that the model is robust on the uncertainties.

Like each study, this study was subjected to some limitations. According to the clinical trials mentioned earlier, the outcomes were published based on for four years follow up. On the other hand, guidelines for cost-effectiveness analyses recommend the long time horizon in pharmacoeconomics studies for chronic diseases such as BPH. Hence, we extrapolated clinical trial outcomes to 10 years. As mentioned above, 70 years old men were selected as the age of base scenario and ten years modeling was reasonable and confirmed by experts (38-40).

Second, the generalizability of results to other health care systems may be limited. The health care costs in developing and developed countries are different. This study is based on Tariff, health care system costs, and drug prices in Iran. Then it should be considered that the calculated costs are not comparable with other countries.

Third, the analysis conducted here has used current prices for pharmacotherapy and surgery; however, it is likely that the costs will change over time, which will affect the cost-effectiveness results.

The major challenge in this study was to estimate and gather model inputs because of the lack of local data in the literature. Clinical data were extracted from overseas clinical trials. Transitional probabilities came from published articles and were verified by the experts. Of course, the low sensitivity of the results to changing various parameters indicates fair robustness of the conclusions of this study. 

## Conclusion

Based on the results of this study, we can expect that surgery would be more cost-effective for patients in younger than 60 years old. So, replacing dutasteride with surgery in moderate BPH in Iranian patients aged 60-70 years old and over 70 years old is recommended. 

Our results may be useful for policymakers to allocate resources efficiently regarding the pharmaceutical treatment of BPH.
